# CCAAT/enhancer-binding protein β promotes receptor activator of nuclear factor-kappa-B ligand (RANKL) expression and osteoclast formation in the synovium in rheumatoid arthritis

**DOI:** 10.1186/s13075-015-0532-6

**Published:** 2015-02-17

**Authors:** Hidetoshi Tsushima, Ken Okazaki, Kohei Ishihara, Takahiro Ushijima, Yukihide Iwamoto

**Affiliations:** Department of Orthopaedic Surgery, Graduate School of Medical Sciences, Kyushu University, 3-1-1 Maidashi, Higashi-ku, Fukuoka 812-8582 Japan

## Abstract

**Introduction:**

CCAAT/enhancer-binding protein β (C/EBPβ) is a transcription factor that is activated in the synovium in rheumatoid arthritis (RA) and promotes expression of various matrix metalloproteinases. In this study, we examined whether C/EBPβ mediates the expression of receptor activator of nuclear factor-kappa-B ligand (RANKL) and drives osteoclast formation in primary fibroblast-like synoviocytes (FLS) from RA patients. The cooperation of C/EBPβ and activation transcription factor-4 (ATF4) in the regulation of the RANKL promoter was also investigated.

**Methods:**

Immunofluorescence staining was performed for C/EBPβ, RANKL, and ATF4 in synovium from RA patients. Adenovirus expression vectors for two major isoforms, C/EBPβ-liver-enriched activator protein (LAP) and - liver-enriched inhibitory protein (LIP), or small interfering RNA for C/EBPβ, were used to manipulate C/EBPβ expression in RA-FLS. RA-FLS over-expressing C/EBPβ were co-cultured with peripheral blood mononuclear cells (PBMCs) to test osteoclast formation by tartrate-resistant acid phosphatase (TRAP) staining. A promoter assay for RANKL, a chromatin immunoprecipitation (ChIP) assay and an immunoprecipitation (IP) assay were also performed.

**Results:**

Immunofluorescence staining showed colocalization of C/EBPβ, ATF4 and RANKL in RA synovium. Western blotting revealed the expression of C/EBPβ-LAP and -LIP in RA-FLS. Over-expression of either C/EBPβ-LAP or -LIP significantly increased the expression of RANKL mRNA, while C/EBPβ-LIP down-regulated osteoprotegerin (OPG) mRNA. The RANKL/OPG mRNA ratio was significantly increased by C/EBPβ-LIP over-expression. Knockdown of C/EBPβ with siRNA decreased the expression of RANKL mRNA. The number of TRAP-positive multinucleated cells was increased in co-cultures of PBMCs and FLS over-expressing either C/EBPβ-LAP or -LIP, but was more significant with LIP. C/EBPβ-LIP does not have a transactivation domain. However, promoter assays showed that C/EBPβ-LIP and ATF4 synergistically transactivate the RANKL promoter. ChIP and IP assays revealed the cooperative binding of C/EBPβ and ATF4 on the RANKL promoter.

**Conclusions:**

We demonstrated that C/EBPβ, especially C/EBPβ-LIP in cooperation with ATF4, is involved in osteoclast formation by regulating RANKL expression in RA-FLS. These findings suggest that C/EBPβ plays a crucial role in bone destruction in RA joints.

**Electronic supplementary material:**

The online version of this article (doi:10.1186/s13075-015-0532-6) contains supplementary material, which is available to authorized users.

## Introduction

Cartilage degeneration and bone destruction are the main features of rheumatoid arthritis (RA) [1]. Inflammation pathways are involved in the catabolic processes of articular cartilage and bone degeneration in RA. Inflammatory cytokines such as IL-1β, TNF-α, IL-6, and IL-17 play significant roles in mediating inflammation and joint destruction. These cytokines are expressed in arthritic joints in RA and induce expression of receptor activator of nuclear factor kappa B ligand (RANKL) in the synovium [2]. RANKL is an essential factor for osteoclast differentiation [3,4]. Osteoprotegerin (OPG) is a decoy receptor that inhibits RANKL activation of osteoclastogenesis and reduces bone resorption [5]. RA synovium-induced RANKL stimulates osteoclast differentiation at sites where bone and RA synovial membranes contact each other.

Inflammatory cytokines in RA joints activate numerous transcription factors including nuclear factor-kappa-B (NF-κB), activator protein-1 (AP-1), janus kinase-signal transducer and activator of transcription (JAK-STAT) and the CCAAT/enhancer-binding protein (C/EBP) family. The C/EBP family consists of six members: C/EBPα, β, δ, ε, γ, and ζ [6]. C/EBPβ is an intron-less gene and has three major isoforms: 38 kD (liver-enriched activator protein Star (LAP*)), 36 kD (LAP) and 20 kD (liver-enriched inhibitory protein (LIP)) [7]. The isoforms, LAP* and LAP, each contain an N-terminal transactivation domain (TAD) and a chromatin remodeling domain. The LIP isoform lacks the TAD, although it retains DNA binding capability, and is generally recognized to be a dominant negative isoform.

Recent studies indicated that C/EBPβ is involved in differentiation of osteoblasts and osteoclasts both physiologically and pathologically. C/EBPβ activates osteocalcin gene transcription and promotes osteoblast differentiation [8-10]. For osteoclast differentiation, the C/EBPβ isoform ratio in mononuclear cells regulates osteoclastogenesis through V-maf musculoaponeurotic fibrosarcoma oncogene homolog B (MafB) [11]. C/EBPβ and RANKL are upregulated in GCT. C/EBPβ induces RANKL promoter activity in GCT stromal cells, which causes osteolysis [12]. In inflammatory chronic diseases such as RA, C/EBPβ is strongly induced in response to inflammatory stimulation. C/EBPβ is expressed in synovial tissues and chondrocytes of RA [13,14]. C/EBPβ plays a crucial role in cartilage degradation along with proteolytic enzymes such as matrix metalloproteinase-1 (MMP-1), MMP-3, MMP-13, and aggrecanase-2 (a disintegrin and metalloproteinase with thrombospondin motifs-5: ADAMTS-5) in inflammatory arthritis. Hence, we hypothesized that an imbalance of C/EBPβ isoforms may upset skeletal integrity in RA by being involved in both cartilage and bone destruction.

In this paper, we investigated whether C/EBPβ mediates the expression of RANKL in RA synovium and consequently, whether it induces osteoclast formation. In addition, we analyzed the mechanism of RANKL and OPG expression by the C/EBPβ isoforms, C/EBPβ-LAP and –LIP, and by cooperation with activation transcription factor-4 (ATF4). Determining the mechanisms related to the regulation of RANKL expression and bone resorption by C/EBPβ may provide new insights into the development of potential therapies for RA patients.

## Methods

### Clinical samples

Tissue samples of synovium were obtained from patients with RA at the time of total knee arthroplasty (TKA) or synovectomy. Patients signed informed consent for providing tissue samples for this study. Subjects included seven RA patients (mean age, 60.3 ± 11.3 years), who fulfilled the 2010 American College of Rheumatology (ACR) and the European League Against Rheumatism (EULAR) diagnostic criteria for RA [15]. All studies were performed under the approval of the Institutional Ethics Board of Kyushu University (approval number: 22–99) and in accordance with the tenets of the Declaration of Helsinki.

### Isolation of human fibroblast-like synoviocytes

Human fibroblast-like synoviocytes were isolated from the synovium of RA patients (RA-FLS). Synovial tissues were minced into small pieces and digested with 2 mg/ml collagenase L (Wako, Osaka, Japan) for 90 minutes at 37°C. The collected cells were resuspended in DMEM supplemented with 10% FBS (Gibco, Gaithersburg, MD, USA). Adherent cells were used after three to five passages.

As a control for RA-FLS, human fibroblast-like synoviocytes (HFLS, Cell Applications, San Diego, California, USA), which is a cell line derived from normal synovial tissue, were also cultured in DMEM supplemented with 10% FBS.

### Immunofluorescence staining

Specimens were incubated overnight at 4°C with primary rabbit polyclonal anti-C/EBPβ antibodies (C-19; Santa Cruz Biotechnology, Santa Cruz, CA, USA) diluted 1:100, mouse monoclonal anti-RANKL antibodies (ab45039; Abcam, Cambridge, England) diluted 1:50, gout polyclonal anti-OPG antibodies (sc-8468; Santa Cruz Biotechnology) diluted 1:100, rabbit polyclonal anti-ATF4 antibodies (sc-200; Santa Cruz Biotechnology) or normal rabbit IgG (sc-2027; Santa Cruz Biotechnology) diluted 1:100, respectively. RA-FLS plated on glass coverslips were transfected with adenovirus expression vectors for C/EBPβ-LAP, −LIP or LacZ control [16] for 24 hours and then replaced with fresh medium. After 48 hours, immunofluorescence staining was performed.

### Treatment of cells with cytokines

Confluent cultures of RA-FLS were subjected to serum-free medium for 24 hours. This medium was replaced with fresh medium containing cytokines as follows: IL-1β (R&D Systems, Minneapolis, MN, USA) at a concentration of 2 ng/ml, TNF-α (Sigma-Aldrich, St Louis, MO, USA) at 10 ng/ml, IL-6 (R&D Systems) at 10 ng/ml, and IL-17 (R&D Systems) at 100 ng/ml. Cells were cultured for a further 48 hours. Concentrations of cytokines were determined based on previous literature [17-19].

### Western blotting

Nuclear proteins were isolated using Nuclear and Cytoplasmic Extraction Reagent (NE-PER; Pierce, Rockford, IL, USA). Protein samples were transferred onto nitrocellulose membranes and were treated overnight at 4°C with primary antibodies.

### RNA extraction and real-time reverse transcription (RT)-PCR

Quantitative RT-PCR was performed with the LightCycler 2.0 system (Roche, Basel, Switzerland) using SYBR Premix Ex Taq (Takara Bio, Ohtsu, Japan). The primers were as follows: for C/EBPβ, 5′-AGTACAAGATCCGGCGCGAG-3′ (sense) and 5′-TGCTTGAACAAGTTCCGCAG-3′ (antisense); for RANKL, 5′-ATGAACTCCTTCTCCACAAGCG-3′ (sense) and 5′-CTCCTTTCTCAGGGCTGAG-3′ (antisense; purchased from Takara Bio; oligo name HA137381F and R); for OPG, 5′-GCTTGAAACATA GGAGCTG-3′ (sense) and 5′-GTTTACTTT GGT GCCAGG-3′ (antisense); for ATF4, 5′-TCAAACCTCATGGGTTCTCC-3′ (sense) and 5′-GTGTCATCCAACGTGGTCAG-3′ (antisense); and for GAPDH, 5′-GGTGAAGGTCGGAGTCAACGGA-3′ (sense) and 5′-GAGGGATCTCGCTCCTGGAAGA-3′ (antisense). Data were normalized to the expression of GAPDH.

### Osteoclast formation in a peripheral blood mononuclear cell (PBMC) and RA-FLS co-culture system

Peripheral blood was obtained from healthy donors. Isolated PBMCs (2 × 10^5^ cells/well) were resuspended in α-minimum essential medium (α-MEM) containing 10% FBS and 50 ng/ml macrophage colony-stimulating factor (M-CSF; R&D Systems) and then seeded in 96-well tissue culture plates. Three days later, adherent cells were used for the co-culture system.

Isolated FLS were transfected with adenovirus expression vectors for 24 hours and then fresh medium containing 10% FBS was added. After 48 hours, FLS were added into the 96-well plate with cultured PBMCs in α-MEM containing 10% FBS and 50 ng/ml M-CSF. After 72 hours of co-culture, wells were stained for tartrate-resistant acid phosphatase (TRAP) (Primary Cell Co, Hokkaido, Japan). Osteoclasts were identified as TRAP-positive multinucleated cells that contained more than three nuclei.

### Gene knockdown in RA-FLS

Predesigned small interference RNA (siRNA) for C/EBPβ (C/EBPβ siRNA-1 target sequence, 5′-CCCACGUGUAACUGUCAGCtt-3′ (sense) and 5′-GCUGACAGUUACACGUGGGtt-3′ (antisense)) or negative-control siRNA was purchased (Ambion, Austin, TX, USA). Transfection mixes were prepared using Lipofectamine 2000 (Invitrogen, Carlsbad, CA, USA). RA-FLS cells were cultured for 24 hours after transfection and then treated with 10 ng/ml IL-1β for 72 hours.

### Human RANKL promoter reporter constructs

Promoter constructs for human RANKL were sub-cloned into the pGL-4.10 (luc2) vector (Promega, Madison, WI, USA). The 5′-upstream region (−1591 bp) of the human RANKL gene was prepared using human genomic DNA as a template (p-full). There are four putative binding sites for C/EBPβ between –1591 bp and +12 bp. A 2-bp mutation (AA to CC) was made at one site on the p-full construct using the QuickChange site-directed mutagenesis kit (Stratagene, La Jolla, CA, USA).

### Plasmid transfection and luciferase assay

HeLa cells seeded in 12-well plates were co-transfected with 0.5 μg/well RANKL promoter constructs and various concentrations of pCMV-LAP, an expression vector of rat C/EBPβ-LAP directed by a cytomegalovirus promoter [20], or pCI-neo-LIP, an expression vector of rat C/EBPβ-LIP [21], or pCMV6-AC-GFP-tagged ATF4 (OriGene, Rockville, MD, USA), an expression vector of human ATF4, using Lipofectamine LTX (Invitrogen). pRL-SV40 (Promega) was used as an internal control. Luciferase activity was then assayed using the Dual-luciferase Reporter Assay System (Promega).

### Chromatin immunoprecipitation (ChIP) assay

RA-FLS cells were transfected with the adenovirus vector C/EBPβ-LIP and incubated for 72 hours. A ChIP assay was performed with a ChIP Assay kit (Upstate Biotechnology, Lake Placid, NY, USA). The primers used in the PCR for RANKL promoter sequences were as follows: 5′-GAGGGCGAAAGGAAGGAAGGGGAG-3′ (sense) and 5′-GGCGTTGGAGAGCCCTGGCCTCGG -3′ (antisense), which amplified between −125 bp and +26 bp. For a negative control, sequence between −1727 bp and −1487 bp was used. The PCR products were amplified for 33 cycles.

### Immunoprecipitation (IP)

Nuclear proteins were isolated from RA-FLS transfected with adenovirus vector C/EBPβ-LIP for 72 hours. The IP protocol used Dynabeads Protein A (Invitrogen). Anti-C/EBPβ antibodies, anti-ATF4 antibodies or normal rabbit IgG and Dynabeads-complex, respectively, were added to antigen-containing lysates. Proteins were separated by SDS-PAGE and immunoblotted using specific antibodies.

### Statistical analyses

For *in vitro* investigations, nonparametric comparisons were performed using the Mann-Whitney *U*-test. *P*-values less than 0.05 were considered significant.

## Results

### Co-localization of C/EBPβ and RANKL in the synovium from RA patients

**Figure 1:**
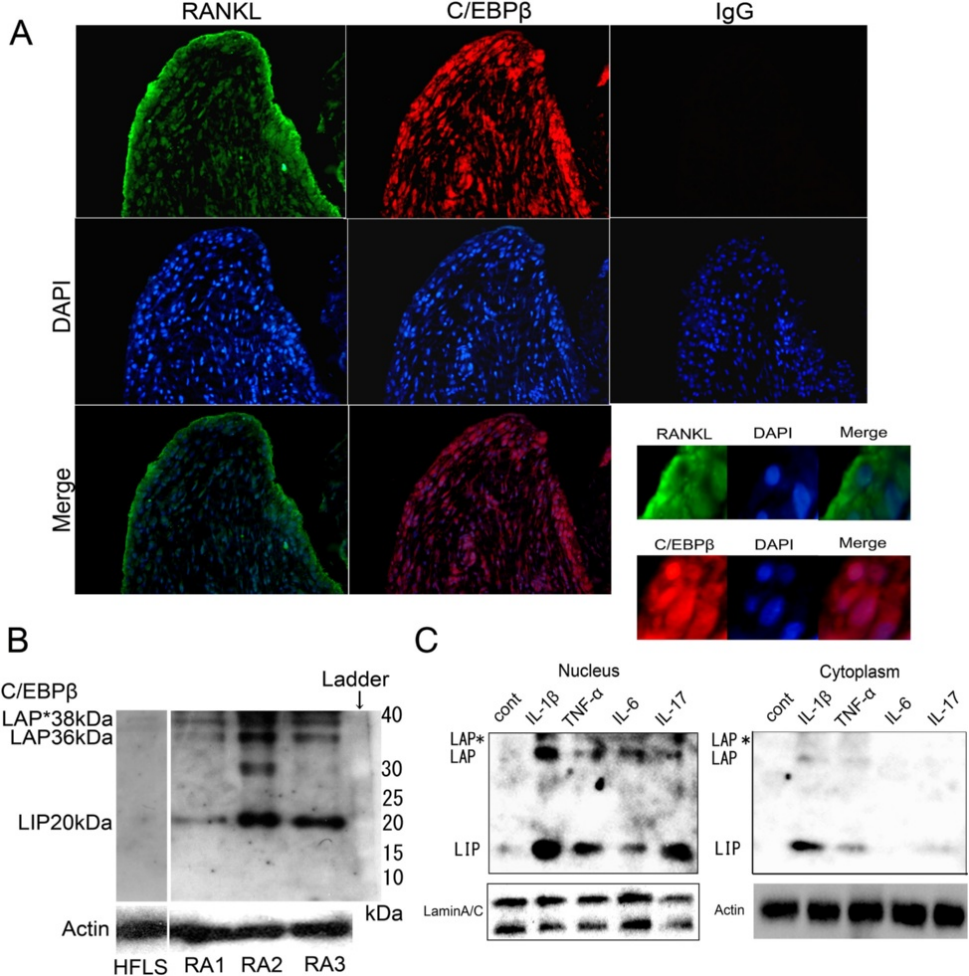
**Expression of C/EBPβ in rheumatoid arthritis (RA) synovium.** **(A)** Expression and distribution of C/EBPβ and RANKL in RA synovium by immunofluorescence staining. RANKL: anti-RANKL antibodies; C/EBPβ: anti-C/EBPβ antibodies; IgG: normal IgG antibodies. The appropriate species Alexa Fluor 488 and 568-conjugated antibodies were used as secondary antibodies and 4′,6-diamidino-2-phenylindole dihydrochloride (DAPI) was applied as a nuclear stain. Original magnification, 200×. Magnified views (600×) at the synovial lining layer are also shown. **(B,** **C)** Western blotting for C/EBPβ protein consisting of 38 kD liver-enriched activator protein (LAP*), 36 kD LAP and 20 kD liver-enriched inhibitory protein (LIP) **(B)** Experiments with whole extracts of primary cultured passage-1 RA fibroblast-like synoviocytes (RA-FLS) from three patients (RA1, RA2 and RA3) and normal human fibroblast-like synoviocytes (HFLS). A band on 30 kD in RA2 might be a non-specific band. **(C)** RA-FLS were treated for 48 hours with IL-1β (2 ng/ml), TNF-α (10 ng/ml), IL-6 (5 ng/ml), or IL-17 (100 ng/ml), respectively. Nuclear extracts and cytoplasmic protein from RA-FLS treated with cytokines were analyzed. Representative data from three independent experiments are shown.

We initially examined C/EBPβ and RANKL expression by immunofluorescence staining in erosive areas of synovial tissue from RA patients. C/EBPβ and RANKL were expressed in RA synovial tissue (Figure 1A). The distribution patterns of C/EBPβ and RANKL were similar and both were strongly expressed in the synovial lining layer rather than in the sub-lining layer. The co-localization of C/EBPβ and RANKL in RA synovium suggests that C/EBPβ is involved in the regulation of RANKL expression.

### Expression of C/EBPβ in RA-FLS after treatment with pro-inflammatory cytokines

Primary cultures of FLS were established and C/EBPβ expression was examined by western blotting. C/EBPβ-LAP* (38 kDa), −LAP (36 kDa) and -LIP (20 kDa) were detected with LIP showing dominant expression. There was a varying degree of C/EBPβ expression (Figure 1B). The difference in expression levels of C/EBPβ may depend on the history of the patients such as degree of inflammation at the time of sample collection, disease duration, or therapies. Human FLS from normal articular joints lacks C/EBPβ protein expression.

Next, we set out to determine whether pro-inflammatory cytokines could promote C/EBPβ protein in FLS. Western blots revealed that stimulation with IL-1β (2 ng/ml), TNF-α (10 ng/ml), IL-6 (5 ng/ml), or IL-17 (100 ng/ml) increased the expression of both LAP and LIP isoforms in nuclear extracts, whereas the samples without any treatment did not show expression of C/EBPβ protein (Figure 1C). Interestingly, the expression of LIP was higher than that of LAP as shown in experiments of primary cultured RA-FLS.

### Overexpression of C/EBPβ regulates expression of RANKL and OPG in RA-FLS

**Figure 2:**
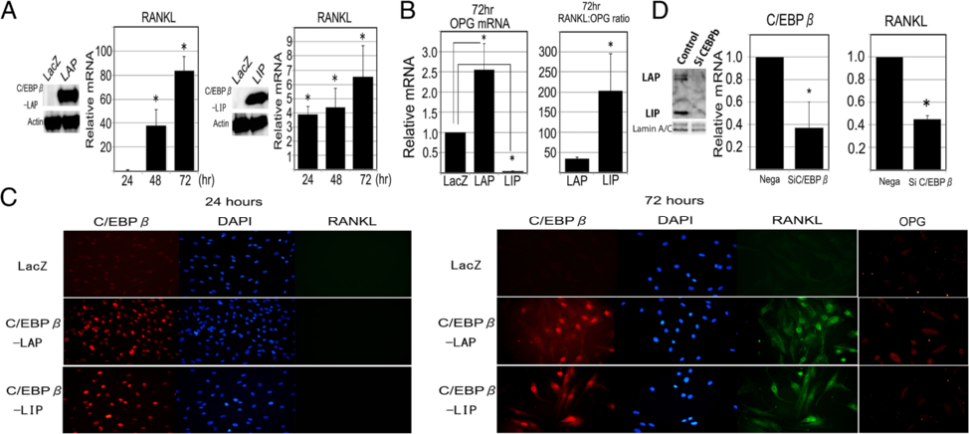
**Gain and loss of function for C/EBPβ in fibroblast-like synoviocytes from the synovium of rheumatoid arthritis patients (RA-FLS).** **(A,** **B)** Effect of C/EBPβ overexpression on RANKL and OPG expression in RA-FLS. RA-FLS were transfected with adenovirus expression vector for C/EBPβ-LAP, −LIP or LacZ (negative control) and cultured for 24 hours. Whole cell extracts were assayed by western blotting for C/EBPβ. RANKL and OPG mRNA expression were analyzed by quantitative RT-PCR from three samples**.** The RANKL-OPG mRNA ratio with the over-expression of C/EBPβ-LAP or -LIP was calculated: *P <0.05 versus control using the Mann-Whitney *U*-test. **(C)** Immunofluorescence staining for RANKL or C/EBPβ protein in RA-FLS with overexpression of C/EBPβ-LAP, −LIP or LacZ control at 24 hours and 72 hours. Immunofluorescense staining for OPG was also performed in a different series of experiments with the same method at 72 hours. Original magnification, 400×. **(D)** Effect of C/EBPβ knockdown on RANKL mRNA expression in RA-FLS. RA-FLS transfected with siRNAs were cultured with 10 ng/ml IL-1β for 48 hours and the expression of C/EBPβ and RANKL mRNA were analyzed by quantitative RT-PCR. The C/EBPβ expression was effectively reduced by siRNA C/EBPβ transfection as confirmed by western blotting for nuclear extracts. RANKL expression was significantly decreased to less than 50%: *P <0.05 versus control using the Mann-Whitney *U*-test.

RA-FLS cells were transfected with adenovirus expression vectors expressing C/EBPβ-LAP, −LIP or LacZ control. Western blots confirmed the exogenous overexpression of LAP or LIP in whole protein extracts isolated from transfected cells (Figure 2A). RANKL mRNA expression was examined by quantitative RT-PCR. The overexpression of LAP induced RANKL mRNA expression up to 80-fold compared to the LacZ control in a time-dependent manner. In RA-FLS transfected with the LIP vector, RANKL mRNA expression was increased approximately 6-fold (Figure 2A). We also investigated the expression of OPG. Expression of OPG mRNA was upregulated by LAP in RA-FLS, whereas LIP significantly reduced OPG mRNA. Consequently, the RANKL-OPG ratio was highly upregulated in RA-FLS transfected with LIP (Figure 2B).

In addition, we examined whether C/EBPβ induced RANKL expression at the protein level by cell fluorescent immunostaining in a time-course experiment. The stimulated expression of C/EBPβ was observed in the nucleus of RA-FLS at 24 hours (Figure 2C). RANKL protein was localized in the cell cytoplasm of FLS over-expressing LAP or LIP at 72 hours. Similarly, the expression of OPG was also examined in a different series of experiments. The expression of OPG was stimulated by C/EBPβ-LAP, but not by C/EBPβ-LIP in RA-FLS at 72 hours.

### C/EBPβ knockdown by siRNA reduced RANKL expression in RA-FLS

We assessed the effect of C/EBPβ knockdown on RANKL mRNA expression using siRNAs targeting C/EBPβ mRNA. Transfected cells were cultured with IL-1β. C/EBPβ knockdown significantly reduced RANKL mRNA expression by 50% after IL-1β treatment in RA-FLS (Figure 2D).

### C/EBPβ induced osteoclast formation through RANKL expression in RA-FLS

**Figure 3:**
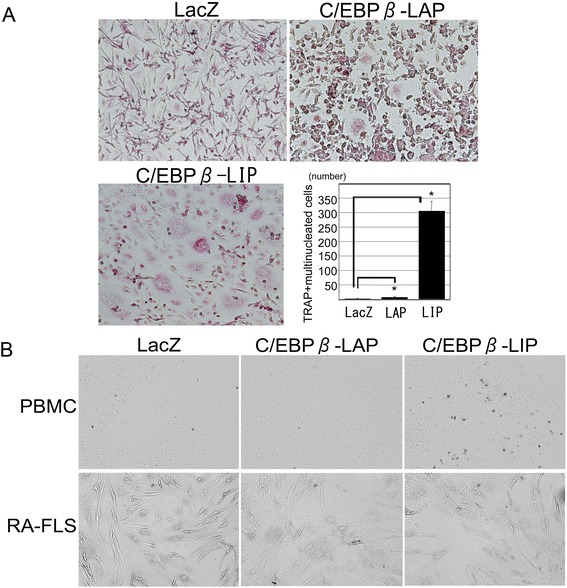
**Osteoclast formation from peripheral blood mononuclear cells (PBMC) is promoted by the enhanced expression of C/EBPβ in fibroblast-like synoviocytes from the synovium of rheumatoid arthritis patients (RA-FLS). (A)** Co-cultures of PBMCs and RA-FLS transfected with adenovirus vectors expressing C/EBPβ-LAP, −LIP or LacZ control for 72 hours. Number of TRAP-positive multinucleated cells recognized in the co-culture system. Osteoclasts were identified as TRAP-positive multinucleated cells that contained more than three nuclei. Original magnification, 100×. *P <0.05 versus control using the Mann-Whitney *U*-test. **(B)** Negative control for the co-culture experiments. PBMC or RA-FLS over-expressing C/EBPβ-LAP, −LIP or LacZ control, respectively, were cultured for 72 hours. No TRAP-positive multinucleated cells were observed.

To investigate whether C/EBPβ-induced RANKL expression stimulated osteoclast formation, we co-cultured RA-FLS, which were transfected with adenovirus vectors expressing C/EBPβ-LAP, −LIP or LacZ control, and PBMCs, which were isolated and stimulated with M-CSF. After three days of co-culture, formation of TRAP-positive multinucleated cells was observed in co-cultures of RA-FLS over-expressing LAP or LIP, but not in control cells. Interestingly, more multinucleated cells were induced in the co-cultures of RA-FLS over-expressing LIP than with LAP transfected cells (Figure 3A). As a negative control, we performed monotype cell cultures of RA-FLS over-expressing C/EBPβ, or PBMC over-expressing C/EBPβ. These cells did not form osteoclasts (Figure 3B).

### C/EBPβ functions as an activator of the human RANKL promoter

**Figure 4:**
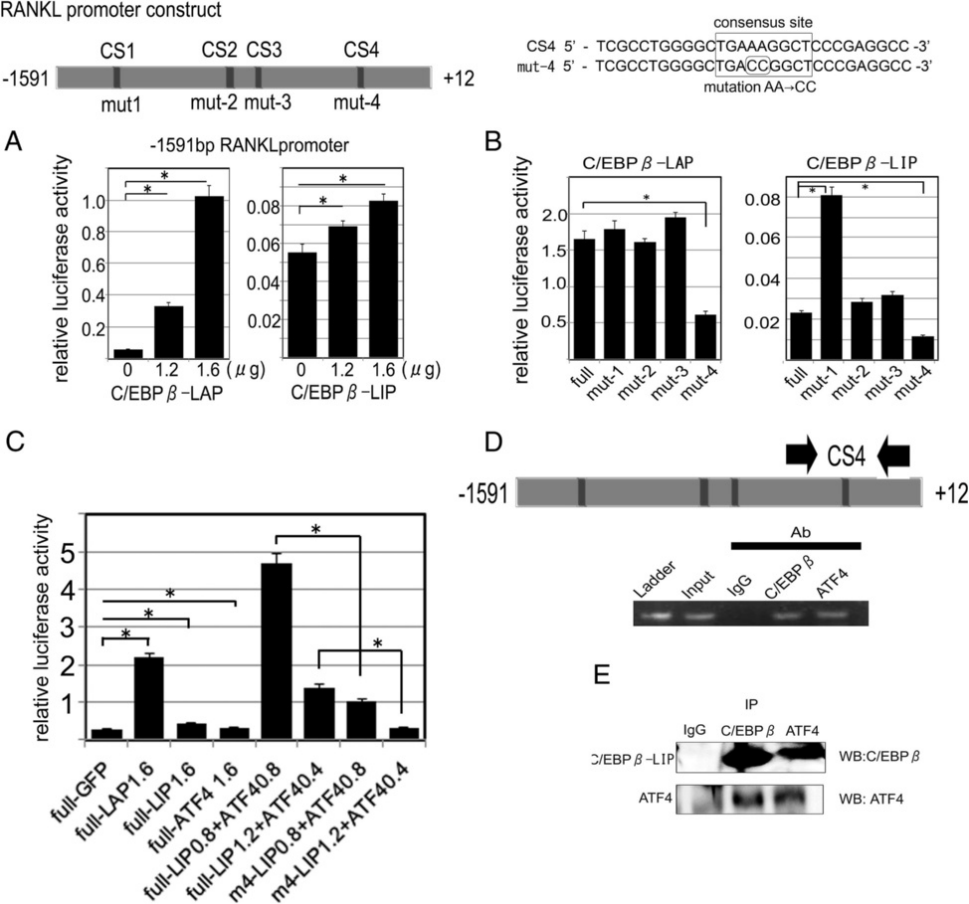
**Transactivation of the human RANKL promoter by C/EBPβ and ATF4.** Schematic of the constructs of the 1603 bp (−1591 bp ~ +12 bp) RANKL promoter, which were subcloned into the pGL-4.10 (luc2) vector. Analysis of the sequence indicated the presence of four C/EBPβ binding motifs (CS1-4). **(A)** The RANKL promoter-luciferase reporter vectors were co-transfected into HeLa cells with pCMV-LAP or pCI-LIP. Relative luciferase activity was assayed 24 hours post-transfection. **(B)** A 2-bp mutation (AA to CC) was made at one site (mut-1, mut-2, mut-3, mut-4). The RANKL promoter mutation constructs were co-transfected with pCMV-LAP or pCI-LIP into HeLa cells. **(C)** Cooperative effect of C/EBPβ and ATF4 on the RANKL promoter. Constructs of the RANKL promoter were co-transfected with expression vectors for C/EBPβ-LAP, −LIP, ATF4 or GFP. Synergistic activation of the promoter was observed with the combination of C/EBP-LIP and ATF4. The effect was diminished when the promoter harbored a mutation at CS4. Full: 1.6 kb human RANKL promoter; m4: 1.6 kb human RANKL promoter that has a mutation at CS4. **(D)** A chromatin immunoprecipitation assay was performed using C/EBPβ or ATF4 specific antibodies or control IgG in RA-FLS after treatment with IL-1β for 48 hours. The area containing the C/EBPβ consensus binding site 4 (CS4) on the RANKL promoter was amplified by semiquantitative RT-PCR. **(E)** Immunoprecipitation was performed using C/EBPβ or ATF4 specific antibodies or control in RA-FLS over-expressing C/EBPβ-LIP. Western blots for C/EBPβ or ATF4 showed a positive band for either C/EBPβ-LIP or ATF4.

We further analyzed the *in vitro* promoter activity of human RANKL using HeLa cells. A luciferase reporter gene construct containing −1591 bp of the RANKL promoter was co-transfected with the expression vectors for C/EBPβ-LAP (pCMV-LAP) or C/EBPβ-LIP (pCI-neo-LIP) into HeLa cells. RANKL promoter activity was upregulated in a dose-dependent manner with either LAP or LIP (Figure 4A). To identify the C/EBPβ responsive element in the RANKL promoter, mutation analysis was performed using site-directed mutagenesis. We created four single mutation constructs in the RANKL promoter: single mutation 1 (mut-1), mutation 2 (mut-2), mutation 3 (mut-3), and mutation 4 (mut-4). Luciferase activities of mut-1, mut-2, and mut-3 reporter constructs were equally increased with pCMV-LAP, while mut-4 reporter construct decreased luciferase activity by 30% (Figure 4B). Similarly, using the C/EBPβ-LIP expression vector, mut-2 and mut-3 did not show difference of activity from full reporter construct, while mut-4 decreased to 25%. These results showed that the putative C/EBPβ binding site is located between −59 bp and −52 bp in the RANKL promoter. The rationale for the increased activity of mut-1 with C/EBPβ-LIP expression vector has not been discovered yet as we failed to show the direct binding of C/EBPβ on this site.

### C/EBPβ-LIP and ATF4 synergistically stimulate RANKL expression

C/EBPβ-LIP does not have an activation domain and is considered to be a dominant negative isoform. However, our results suggest that LIP is involved in RANKL expression in RA-FLS and plays a role in induction of osteoclast formation. We hypothesized that some transcriptional co-factors may cooperate with C/EBPβ-LIP to activate transcription of the RANKL promoter. ATF4 is known to stimulate RANKL expression in osteoblasts [22]. Additionally, ATF4 has been shown previously to interact with C/EBPβ, which activates various downstream factors such as osteocalcin and discoidin domain receptor tyrosine kinase (DDR2) [23]. Thus, we considered that a similar mechanism might exist for the regulation of RANKL gene expression. A luciferase assay showed that ATF4 slightly activated the RANKL promoter (Figure 4C). RANKL promoter activity was significantly enhanced following co-transfection of LIP and ATF4. Mut-4 abrogated the responsiveness of the RANKL promoter to the combination of LIP and ATF4. A ChIP assay was performed using RA-FLS over-expressing LIP and primers constructed from the human RANKL promoter sequence, which amplify sites including the C/EBPβ consensus site-4. This analysis indicated that LIP binds to the RANKL promoter region containing CS-4 and that ATF4 also binds in the same region (Figure 4D). IP and immunoblotting demonstrated that ATF4 bound to over-expressed LIP in RA-FLS (Figure 4E). Collectively, these results suggest that C/EBPβ-LIP cooperates with ATF4 in activating RANKL gene expression.

### ATF4 constitutively exists in RA synovium

**Figure 5:**
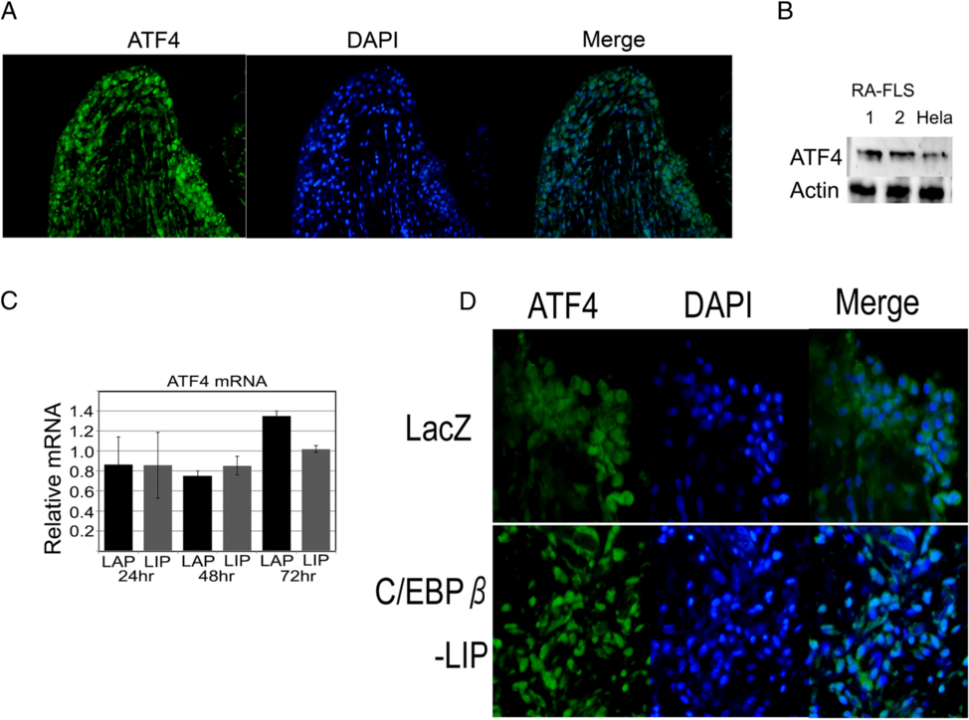
**ATF4 is constitutively expressed in rheumatoid arthritis (RA) synovium, but translocated into the nucleus by C/EBPβ-LIP.** **(A)** Expression and distribution of ATF4 in RA synovium by immunofluorescence staining. Original magnification, 200×. **(B)** Expression of ATF4 protein levels from fibroblast-like synoviocytes from the synovium of RA patients (RA-FLS) by western blotting. ATF4 protein in HeLa cells was used as a positive control. **(C)** Expression of ATF4 mRNA of RA-FLS overexpressed C/EBPβ by quantitative RT-PCR. Isolated RA-FLS were transfected with adenovirus expression vectors for C/EBPβ-LAP, −LIP or LacZ control. The level of ATF4 mRNA relative to LacZ control is shown. **(D)** Synovial tissue from RA patients was cultured in serum-free medium for 24 hours and then replaced with fresh medium containing adenovirus expression vectors for C/EBPβ-LIP or LacZ control for a further 72 hours. Immunostaining for ATF4 was performed. Original magnification, 400 × .

We then examined the localization of ATF4 in RA synovium. ATF4 was observed in erosive areas of RA synovium by immunofluorescence staining (Figure 5A). Western blotting showed that ATF4 was expressed in whole cell extracts of RA-FLS (Figure 5B). Next, we examined whether ATF4 expression was affected by C/EBPβ in RA-FLS transfected with adenovirus expression vectors in time-course experiments. ATF4 mRNA expression was not significantly changed by C/EBPβ (Figure 5C). In addition, we performed organ cultures using RA synovium tissue. In RA synovium transfected with adenovirus LacZ control, ATF4 mainly localized in the cytoplasm of cells. Interestingly, in RA synovium over-expressing LIP, ATF4 was mainly located in the nucleus (Figure 5D). The results suggest that ATF4 is translocated from the cytoplasm into the nucleus in the RA synovium overexpressing C/EBPβ-LIP although the mechanisms are not clear.

## Discussion

In this study, we have shown that the transcription factor C/EBPβ promotes the expression of RANKL in RA synovium. C/EBPβ-induced RANKL in synovium could induce the formation of osteoclasts. This paper demonstrates that RA-FLS expressed the C/EBPβ-LIP isoform more dominantly than the C/EBP-LAP isoform in response to pro-inflammatory cytokines. C/EBPβ-LIP stimulated RANKL expression even though C/EBPβ-LIP lacks the transactivating domain. Recruitment of ATF4, which is constitutively expressed in the cytoplasm of RA-FLS, to the RANKL promoter might be the mechanism by which C/EBPβ-LIP activates the promoter. More interestingly, the effect of C/EBPβ-LIP in osteoclast formation is stronger than that of C/EBPβ-LAP. The lower expression of OPG might be involved in this mechanism. These results suggest that C/EBPβ-LIP is one of the key regulators of inflammation-induced osteoclast formation. As C/EBPβ is also involved in cartilage degradation [19], C/EBPβ may play a crucial role in joint destruction in RA.

Understanding the mechanisms that mediate RANKL gene expression may aid development of new therapies for reducing bone resorption in RA. We showed that LAP and LIP directly bind a site located between −59 bp and −52 bp of the RANKL promoter. LIP is increased in the RA synovium and forms a complex with constitutively expressed ATF4. This complex may activate transcription of RANKL by binding to the C/EBPβ binding motif of the RANKL promoter. ATF4 belongs to the ATF/cAMP responsive element binding protein (CREB) family, which contains a basic leucine zipper region, and is one of the major regulators of osteoblast differentiation [24]. Moreover, ATF4 regulates RANKL expression [22]. This study showed that ATF4 is expressed in RA synovium and is involved in RANKL expression. Interestingly, in *ex vivo* experiments (Figure 5D), ATF4 was mainly expressed in the cytoplasm of FLS transfected with the LacZ adenovirus vector (control), while ATF4 tended to be located in the nucleus of FLS that overexpressed LIP. ATF4 in cooperation with C/EBPβ might be a crucial regulator of RANKL expression in mediating synovium-induced bone resorption in RA. Other transcription factors, such as NF-κB, AP-1, STAT3 and Runt-related transcription factor-2 (Runx2), may also interface with C/EBPβ. Runx2 and C/EBPβ cooperatively promote the expression of Indian Hedgehog in hypertrophic chondrocytes [25]. STAT3 is induced by IL-1β, TNF-α, and IL-6 and increases the expression of IL-6 and RANKL. A positive feedback loop, via IL-6 and STAT3, enhanced RANKL expression and osteoclastogenesis in inflammatory arthritis [26]. C/EBPβ was previously known as nuclear factor for IL-6 expression (NF-IL6) [27]. IL-6 induced by C/EBPβ regulates C/EBPβ gene transcription with recruitment of STAT3 to the promoter of the C/EBPβ gene, especially in hepatocytes [28]. Therefore, we consider that positive feedback loops involving pro-inflammatory cytokines, IL-6, STAT3 and C/EBPβ, might strongly increase RANKL expression in joints with RA. Indeed, STAT3 is essential for stimulation of RANKL and its binding element is located at −82 bp of the RANKL promoter, which is next to the C/EBPβ responsive motifs. C/EBPβ-LIP may exist as an anchor to form a complex with other transcription factors mediated by inflammatory pathways.

The C/EBPβ isoform ratio can alter in response to cellular processes [7,29,30]. Of note, the LAP-LIP ratio is significant for osteoclastogenesis in PBMC through the mTOR pathway [11]. These papers indicate that an appropriate LAP-LIP ratio results in higher transcriptional activation of the target gene, which is very important in proliferation and differentiation. We showed that LIP protein is more highly expressed in RA-FLS than LAP. This imbalance of the LAP-LIP ratio caused a concomitant change in OPG mRNA expression. OPG is an endogenous inhibitor of RANKL-RANK interaction and is produced in synovial cells of patients with RA. The balance between levels of RANKL and OPG (RANKL-OPG ratio) is correlated with the extent of bone resorption in RA joints [31]. The current study demonstrates that LIP drastically increased the RANKL-OPG ratio in RA-FLS, which subsequently induced significant osteoclast formation.

Previous studies showed that C/EBPβ-LAP is a key regulator of cartilage degradation in inflammatory arthritis. C/EBPβ-LAP plays a crucial role in cartilage degradation along with proteolytic enzymes such as MMP-1, MMP-3, MMP-13, and aggrecanase-2 (ADAMTS-5) in chondrocytes and FLS in inflammatory arthritis [19,32,33]. The role of LIP is not well investigated in inflammatory arthritis. Our unpublished data revealed that overexpression of LIP in FLS increased MMP-1, MMP-3, MMP-9, MMP-13, and ADAMTS-4 mRNA similar to the overexpression of LAP. The data presented here suggest that LAP and LIP coordinate in enhancing expression of RANKL, MMPs, and ADAMTSs, which may result in cartilage degradation and bone destruction of RA joints. C/EBPβ may be a common regulator, which can be stimulated in response to pro-inflammatory cytokines and upregulated in RA synovium. Therefore, selective blockage of C/EBPβ expression may be one potential strategy for preventing inflammation and bone resorption in arthritis.

This study has several limitations. First, double staining for C/EBPβ and RANKL did not work well on the RA synovium sections, although the reason was unclear. Therefore, we stained C/EBPβ and RANKL separately. However, the distribution of these molecules overlapped each other, suggesting that C/EBPβ and RANKL are co-expressed. Next, the promoter assays were performed in HeLa cells. We could not obtain reliable results of the promoter assay in RA FLS probably because of low transfection efficiency. However, the purpose of these experiments was to see the effect of various transcription factors that were exogenously introduced by expression vectors. Therefore, the influence of cell characteristics on the results is considered to be limited. Thirdly, the promoter that harbors a mutation in CS1 showed increased activity with C/EBPβ-LIP (Figure 4B). This result suggests that LIP act as a repressor in CS1. However, ChIP assay for CS1 sequences did not show binding of C/EBPβ on CS1 (data not shown). Therefore, the function of CS1 remains unclear at the moment.

## Conclusions

**Figure 6:**
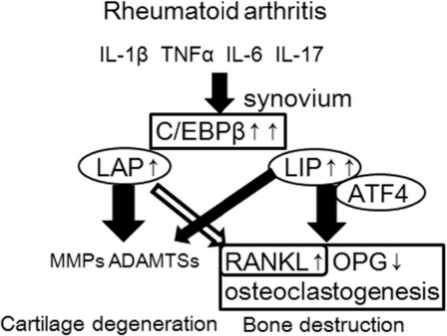
**Scheme of the involvement of C/EBPβ-LAP and -LIP for cartilage and bone destruction in rheumatoid arthritis (RA).** Pro-inflammatory cytokines stimulate expression of C/EBPβ-LAP and -LIP in RA synovium. Synovium over-expressing C/EBPβ, particularly LAP, caused cartilage degeneration through MMPs and ADAMTSs. C/EBPβ-LIP promoted osteoclast formation through stimulation of RANKL and repression of OPG leading to bone destruction. ATF4 cooperates with C/EBPβ-LIP to stimulate RANKL expression.

In conclusion, C/EBPβ increased RANKL expression in RA-FLS and induced osteoclastogenesis (Figure 6). Pro-inflammatory cytokines significantly induced C/EBPβ-LIP, which strongly induced osteoclastogenesis by increasing the RANKL-OPG ratio in RA-FLS. LIP possesses transactivation activity for the RANKL promoter by recruiting ATF4, which constitutively exists in the cytoplasm of RA-FLS, to the C/EBP binding site in the RANKL promoter. In pathological inflammatory arthritis, C/EBPβ is a crucial factor in damaging cartilage and bone in joints.

## Abbreviations

α-MEM: α-minimum essential medium
AP-1: activator protein-1
ATF-4: activation transcription factor 4
bp: base pairs
C/EBP: CCAAT/enhancer binding protein
ChIP: chromatin immunoprecipitation
DMEM: Dulbecco’s modified Eagle’s medium
FBS: fetal bovine serum
FLS: fibroblast-like synoviocytes
GCT: giant cell tumor
IL: interleukin
JAK-STAT: janus kinase-signal transducer and activator of transcription
LAP: liver-enriched activator protein
LIP: liver-enriched inhibitory protein
M-CSF: macrophage colony-stimulating factor
MMP: matrix metalloproteinase
NF-κB: nuclear factor-kappa-B
OPG: osteoprotegerin
PBMC: peripheral blood mononuclear cell
RA: rheumatoid arthritis
RA-FLS: fibroblast-like synoviocytes from the synovium of RA patients
RANKL: receptor activator of nuclear factor kappa B ligand
RT: reverse transcription
siRNA: small interference RNA
TNF: tumor necrosis factor
TAD: N-terminal transactivation domain
TRAP: tartrate-resistant acid phosphatase
